# Mapping China's sports tourism projects: regional imbalances, urban agglomerations, and driving forces

**DOI:** 10.3389/fspor.2026.1761223

**Published:** 2026-03-04

**Authors:** Fanxiang Zhao, Joonyoung Han

**Affiliations:** Department of Kinesiology, Yeungnam University, Gyeongsan, Republic of Korea

**Keywords:** agglomeration economy, geographic detector, kernel density estimation, regional imbalance, spatial autocorrelation, sports tourism

## Abstract

**Introduction:**

The spatial distribution of sports tourism projects in China exhibits significant regional disparities, creating challenges associated with efficient resource utilisation and equitable development.

**Methods:**

In this study, we applied kernel density estimation, spatial autocorrelation analysis, and the geographic detector to examine the spatial patterns and driving factors of STPs across the country systematically.

**Results:**

The results reveal an uneven distribution of STPs, with the Yangtze River Delta and Beijing-Tianjin-Hebei region identified as the two core areas with the highest density. Natural STPs are more widely distributed, whereas cultural STPs are primarily concentrated in economically developed regions. Factor detection reveals that economic and fiscal variables serve as dominant drivers and that the balance of deposits in financial institutions, general public budget expenditures, and tertiary industry operating income exert the greatest explanatory power, whereas transportation and environmental indicators are less influential.

**Discussion:**

These findings highlight the combined effects of natural endowments, cultural resources, and institutional support on sports tourism development. This study advances the theoretical understanding of STP spatial dynamics and provides practical insights for optimising resource allocation, narrowing regional disparities, and promoting the sustainable growth of China's sports tourism industry.

## 1 Introduction

Sports tourism is an emerging green health-oriented industry that integrates sports and tourism (1). Sports tourism has become an important component of the global tourism industry, with significant potential to stimulate economic growth, enhance community engagement, and support sustainable development. The integration of sports and tourism enriches visitor experiences while generating local economic benefits through employment creation and infrastructure investment (2). This integration encompasses both sporting events and recreational activities that can strategically maximise tourism outcomes (3).

Sustainability is central to sports tourism development, requiring a balance between economic gains and social and environmental responsibilities. The concept of sustainable sports tourism highlights the importance of management frameworks that embed sustainability principles to ensure positive contributions to local economies and ecosystems (4, 5). Empirical evidence from Central Africa suggests that, despite substantial development potential, effective trust and cooperation between governments and the private sector are critical for achieving sustainable sports tourism outcomes (6).

Technological advances and data analytics are increasingly shaping sports tourism research and practice. Machine learning techniques have been applied to classify and evaluate ethnic traditional sports tourism, reflecting a shift toward data-driven analytical approaches (7). Similarly, neural network–based models have been developed to improve the accuracy of sports tourism demand forecasting, providing scientific support for industry planning and development (8).

Moreover, the rapid expansion of the internet and social media has intensified scholarly attention on sports tourism marketing. Social media enhances destination attractiveness, brand image, and visitor engagement by fostering emotional connections and encouraging participation (9). Destination management organisations further influence sports tourists through social media content that delivers informational, recreational, and participatory value, which can stimulate booking intentions and positive word-of-mouth behaviour (10).

Research on the spatial patterns of sports tourism in China has gradually evolved into a systematic body of work. Existing literature has revealed both the spatial distribution characteristics of sports tourism resources and mechanisms that drive them. For example, Zuo et al. (11) employed the nearest-neighbour index, kernel density analysis, and a geographic detector model to demonstrate that China's sports tourism signature exhibits exhibit an overall pattern of “urban reliance and proximity to nature”, with high-density clusters emerging in regions such as the Yangtze River Delta. This spatial distribution is shaped by the combined influences of natural endowments, transportation conditions, industrial support, and policy guidance. Further research focusing on the Yangtze River Delta has shown that the spatial configuration of sports tourism resources has undergone a process of “agglomeration–dispersion–re-agglomeration”, with the dominant drivers shifting from “policy and transportation” in the early stages to “economic and social factors”, and eventually to a multi-factor, interactive mechanism (12). Simultaneously, new methodological approaches integrating data mining and fusion techniques have been applied to analyse spatial characteristics, revealing significant clustering in fitness-oriented destinations (13). At the urban level, different cities exhibit diverse developmental pathways depending on their respective foundations and resource endowments. Xiamen has promoted the integration of sports facilities with commercial services through the public–private partnership model, Ningbo has optimised spatial structures to enhance the utilisation of existing facilities, Xinyu has adopted an event-led development approach, and Zhangjiakou has leveraged Winter Olympic facilities to stimulate regional revitalisation (14). Furthermore, studies based on provincial panel data indicate that the overall efficiency of sports–tourism integration in China remains relatively low, with the eastern regions significantly outperforming central and western provinces. These dis-parities are primarily attributable to differences in industrial structure, transportation infrastructure, openness to external markets, and policy support (15).

The 14th 5-Year Plan for Sports Development explicitly calls for the promotion of two development models, namely “tourism + sports” and “sports + tourism”, by establishing representative sports tourism routes, events, and pilot zones to foster the high-quality growth of the industry (16). Despite this rapid expansion, the spatial distribution of sports tourism remains highly uneven, with significant disparities between the eastern and western regions, and between urban and rural areas (11). These imbalances impede the efficient allocation of resources and intensify regional inequalities in tourism-driven economic benefits. Therefore, it is imperative to examine the spatial distribution of STPs. Such an analysis can help uncover the mechanisms underlying regional disparities and provide an empirical foundation for policymakers to optimise resource allocation, promote balanced regional development, and support the sustainable growth of the sports tourism industry.

Agglomeration economies, which have long been emphasised in economic geography and urban studies, refer to the benefits that firms, industries, and institutions derive from being located in close proximity to one another. When applying this concept to sports tourism, it can be argued that urban clusters and regional hubs facilitate the concentration of STPs by enhancing accessibility, attracting skilled labour, and enabling the efficient sharing of supporting services and facilities. Therefore, understanding the interplay between ag-glomeration economies and regional attributes is essential for explaining the spatial patterns and regional disparities in STPs in China.

Contemporary research has investigated the role of agglomeration economies in the tourism sector, emphasising both productivity enhancement and spatial spillover effects. Kim et al. (17) employed spatial econometric methods to demonstrate that tourism-related agglomeration significantly boosts productivity within a region and exerts positive spillover effects on adjacent areas. In China, Wang and Ma (18) revealed a U-shaped relationship between tourism industry agglomeration and total factor productivity in urban agglomerations moderated by public health events such as epidemics. Furthermore, Zhou and Lin (19) highlighted that while tourism agglomeration can drive industrial upgrading and associated environmental benefits, it also poses the risks of congestion and energy–carbon trade-offs.

Amid the rapid growth of sports tourism, the theoretical perspective of agglomeration economies provides a valuable framework for interpreting the uneven spatial distribution of STPs.

This study systematically examined the spatial distribution and aggregation characteristics of STPs across China from a geographical perspective. We investigated regional variations in the STP distribution, assessed the density and degree of aggregation within each area, and evaluated the presence of spatial autocorrelation. Furthermore, we explored the factors influencing the spatial arrangement of STPs at the prefecture level in China. Our objective was to provide a scientific basis for the rational management and allocation of STPs, reduce resource wastage and overdevelopment, enhance the efficiency of resource utilisation and economic returns, and offer theoretical guidance to local authorities for formulating effective sports tourism development strategies.

## 2 Method

### 2.1 Kernel density estimation

Kernel density estimation is a widely used geographical method for studying the concentration of point pattern data (47). The core concept is to consider each sample point as a centre and use a kernel function to measure the contribution of that sample point to other points. Kernel density estimation is widely applied in various fields such as agricultural development (48) and traffic accident monitoring (49). These applications enhance both the precision and effectiveness of data analysis while also offering a scientific foundation for associated decision making, which holds considerable practical significance. This study employed the ArcGIS 10.8 software to perform kernel density estimation calculations aimed at analysing the spatial clustering attributes of natural and cultural STPs. A higher kernel density estimation value indicates a higher degree of STP aggregation. The calculation method for kernel density estimation is shown in Equation 1:

$${\hat{\;f}}_{h}(x)=\frac{1}{nh}\overset{n}{\underset{i=1}{\sum }}\;K\left(\frac{x-X_{i}}{h}\right)$$ (1)

As shown in the table, *K* represents the kernel function, *h* represents the bandwidth, and *n* represents the number of sample points.

### 2.2 Spatial autocorrelation analysis

Spatial autocorrelation reflects the degree of correlation between a certain phenomenon in a region and the same phenomenon in adjacent regional units and is used to determine the potential interdependence between the phenomena (20). It is mainly divided into positive and negative correlations. Positive correlation indicates that the data shows an aggregated state in space, while negative correlation indicates that the data shows a dispersed state in space. Spatial autocorrelation analysis is widely used in geography (21), ecology (22), and medicine (23). It is an important tool in spatial statistics and geographic information systems, helping researchers identify and understand the distribution characteristics of spatial data and providing a scientific basis for decision making.

In this study, spatial autocorrelation analysis was conducted using three complementary methods, namely Moran's *I*, the Getis–Ord General G, and Anselin Local Mo-ran's *I*, to investigate the overall spatial patterns, clustering tendencies, and local hotspots of STPs in prefecture-level cities in China, providing a comprehensive assessment of spatial dependence and heterogeneity. Data processing was conducted using ArcGIS 10.8 software.

#### 2.2.1 Moran's *I*

Moran's *I* is calculated by measuring the degree of mutual correlation between observed values to determine whether the data shows an aggregated or dispersed pattern in the overall space. This study calculates Moran's *I* to assess the state of STR in China in the overall space. Data processing was conducted using ArcGIS 10.8 software. The calculation method is shown in Equation [2].

$$I=\frac{n\sum_{i=1}^{n}\;\sum_{\;j=1}^{n}\;\omega_{ij}(P_{i}-P_{\mathrm{mean}})\times (P_{j}-P_{\mathrm{mean}})}{\left(\sum_{i=1}^{n}\;\sum_{\;j=1}^{n}\;\omega_{ij}\right)\times \sum_{i=1}^{n}\;{\left(P_{i}-P_{\mathrm{mean}}\right)}^{2}}$$ (2)

As shown in the table, $P_{i}$ and $P_{j}$ are the numerical values of the related index for the *i*-th and *j*-th regions, *n* is the number of regions; $P_{\mathrm{mean}}$ is the average value of the index; and $\omega_{ij}$ is the spatial weight matrix (20). The value of Moran's *I* ranges from −1 to 1. When the value is −1, it indicates that the variable is completely dispersed in the overall space; when the value is 0, it indicates that the variable is randomly distributed in the overall space; and while the value is 1, it indicates that the variable is completely aggregated in the overall space.

#### 2.2.2 Getis-Ord General G

Getis-Ord General G is a overall statistic used to assess whether there is a overall high-value or low-value clustering in the entire study (24). Specifically, it calculates the observed and expected values to determine whether the data values are randomly distributed or form clusters in space. In this study, the observed and expected values of STR in the overall space are calculated to determine the characteristics of clustering (25). Data processing was conducted using ArcGIS 10.8 software. The calculation method is shown in Equation 3.

$$G=\frac{\sum_{i}\;\sum_{j}\;w_{ij}x_{i}x_{j}}{\sum_{i}\;x_{i}}$$ (3)

As shown in the table, $x_{i}$ is the observed value of the *i*-th spatial unit, and $w_{ij}$ is the spatial weight matrix.

#### 2.2.3 Anselin Local Moran's *I*

Unlike Moran's *I*, Anselin Local Moran's *I* can reveal the clustering patterns of spatial data in certain regions, helping to identify the patterns like high-value clusters, low-value clusters, or outliers. In this study, Anselin Local Moran's *I* is calculated to explore high and low clustering areas at the municipal level in China. Data processing was conducted using ArcGIS 10.8 software. The calculation method is shown in Equation 4 (26):

$$I_{i}=\frac{\left(x_{i}-\bar{x}\right)}{m_{2}}\underset{j}{\sum }\;w_{ij}z_{j}$$ (4)

As shown in the table, $x_{i}$ is the observed value of the *i*-th spatial unit, $z_{j}$ is the standardized observed value of the *j*-th spatial unit, $w_{ij}$ is the spatial weight matrix, and $m_{2}$ is the variance of the observed values.

### 2.3 Geographic detector

The Geographic Detector is based on the principle of spatial heterogeneity to determine the driving force of a response variable (27). It assumes that the spatial distributions of two variables should be similar if the independent variable shows a significant impact on the dependent variable (28). A key advantage of the Geographic Detector is its non-reliance on linear assumptions and resilience to multicollinearity, making it suitable for socioeconomic and health research (29). This study uses the factor detector in The Geographic Detector to calculate the *q*-value to assess the contribution of socioeconomic indicators to the quantity of STR. The calculation method is shown in Equation 5:

$$q=1-\frac{\sum_{h=1}^{n}\;N_{h}\sigma_{h}^{2}}{N\sigma^{2}}$$ (5)

As shown in the table, *h* = 1,2,…,*n*, which represents the stratification of the independent variable; *N* is the number of regional units; and σ^2^ is the total variance of the study area. When *p* < 0.01, the *q*-value is considered statistically significant (30). The free software for the Geographic Detector can be accessed at http://www.geodetector.cn/ (accessed on 21 August 2025).

### 2.4 Data sources

Data on STPs were obtained from the Sports Culture Development Centre of the General Administration of Sport in mainland China, which published the 2013–2024 China Sports Tourism Signature Projects (https://www.sport.gov.cn/whzx/n5588/c25757858/content.html) (accessed 31 January 2025). The data include scenic spots, events, routes, and destinations. The latitude and longitude coordinates of the projects were obtained using the Baidu Coordinate Picker. To derive coordinates, scenic spots are searched by their names, events are searched by the venue or location name, routes are searched by the starting point name, and destinations are searched by the local government name.

In this study, STPs were classified into natural and cultural categories based on their primary dependency conditions. Natural STPs depend predominantly on ecological and geographical factors such as mountain skiing, coastal recreation, and water sports, and their spatial distribution is largely constrained by natural endowments. In contrast, cultural STPs are rooted in historical traditions, cultural practices, and social institutions, including marathons, traditional sports festivals, and heritage-related sporting events, and tend to cluster in areas characterised by strong cultural identity and institutional support. This classification not only reflects the practical logic underlying sports tourism development but also provides a more nuanced basis for analysing spatial patterns and their driving factors. For example, the Beijing Jundushan Ski Resort, Yanmen Pass National Hiking Trail, and Suqian Santai Mountain Forest Park are classified as natural STPs, while the Shanghai International Marathon, Jiaozuo Taiji Sports Centre, and Yantai International Martial Arts Festival are classified as cultural STPs. STPs that combine natural and cultural attributes are uniformly classified as cultural STPs, such as the Taining World Chinese Mountain Marames and Sichuan Emei Mountain Martial Arts Festival. Repeated projects and projects with different names but at the same location were removed. The final collection consisted of 1,148 coordinate points, including 709 natural and 439 cultural STPs.

Spatial vector data were obtained from the China Resource and Environment Science Data Centre (https://www.resdc.cn/Default.aspx) (accessed 20 September 2024). Data on influencing factors were obtained from the China City Statistical Yearbook and statistical yearbooks of various autonomous prefectures. The collected dataset covers 296 prefecture level cities in mainland China. The influencing factors include Permanent resident population, Urbanisation rate, Number of students enrolled in higher-education institutions, Per capita GDP, GDP growth rate, Proportion of tertiary industry, Operating income of tertiary industry, Total road mileage, Road density, Total public transport passenger volume, General public budget expenditure, Balance of deposits in financial institutions, Proportion of days with good air quality and Green space area. For more detailed data, please refer to the Appendix.

## 3 Results

### 3.1 Spatiotemporal distribution pattern of STPs in China

From a temporal perspective, the number of STPs in China has increased continuously. The total number rose from 90 in 2013 to 1,148 in 2024. The most pronounced growth occurred between 2013 and 2017, with an increase of 443 projects, whereas the smallest increase was observed during 2020–2024, with 239 newly added projects. This slower growth does not indicate a decline in the annual selection of STPs but rather reflects the exclusion of duplicated points of interest (POIs) in the data collection process.

In 2013, STPs in China were mainly concentrated in Guizhou and Anhui provinces, with relatively sparse distribution in other regions. By 2017, a clear nationwide expansion was observed, with a substantial increase in STPs across most provinces. This spatial diffusion indicates that local governments gradually began to attach greater importance to sports tourism and actively promote the development of region-specific STPs.

**Figure 1:**
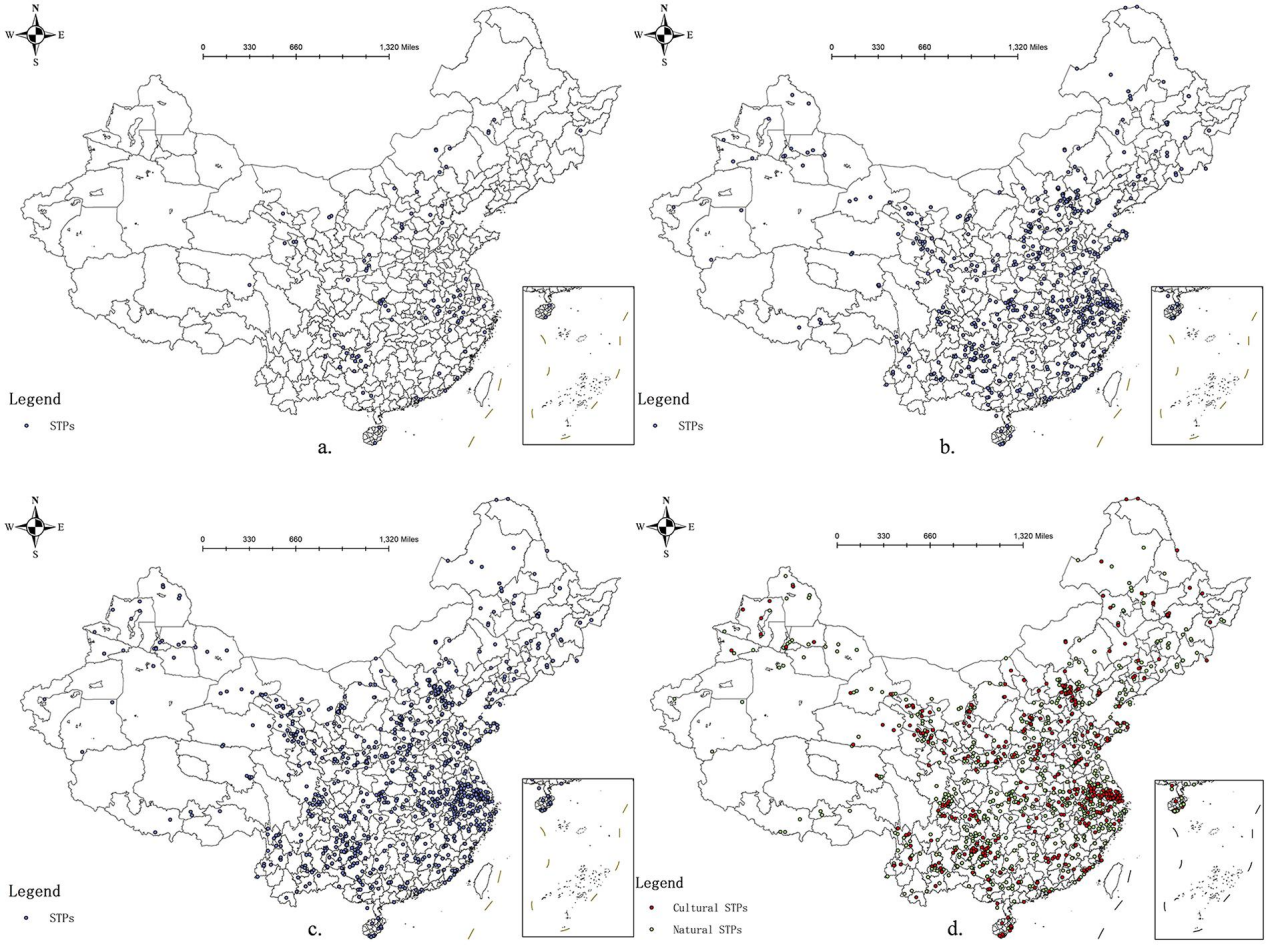
STPs in China: spatial distribution, 2013 **(a)**; 2017 **(b)**; 2020 **(c)**; 2024 **(d)**.

Current, STPs in China are more concentrated in the eastern and southern regions, whereas the western and northern regions have relatively fewer projects (see Figure 1d). The eastern coastal region has 414 projects, accounting for 36.62% of all projects while the central region has 228 projects, accounting for 20.46% of all projects. From a regional perspective, the Yangtze River Delta region, which accounts for only 3.7% of China's land area, contains 19.82% of STPs, making it the densest region of STPs in China. The three provinces of Yunnan, Guizhou, and Sichuan have 169 projects, making them the regions with the largest number of STPs in China. At the provincial level, Jiangsu, Guizhou, and Anhui are the three provinces with the most STPs at 81, 74, and 64 projects, respectively. Tibet has the fewest projects to date.

The spatial distribution pattern of STPs in China is closely related to the “Tengchong-Heihe Line”, which is a population demarcation line in China. Sports tourism projects are highly concentrated in the east, whereas they remain relatively scarce in the west. This contrast underscores the decisive roles of population size, urbanisation, and economic development in shaping the geography of STPs. Furthermore, this imbalance reflects deeper disparities in economic capacity, infrastructure provision, and institutional support, revealing development gaps that market forces alone cannot address.

### 3.2 Spatial distribution density of STPs in China

**Figure 2:**
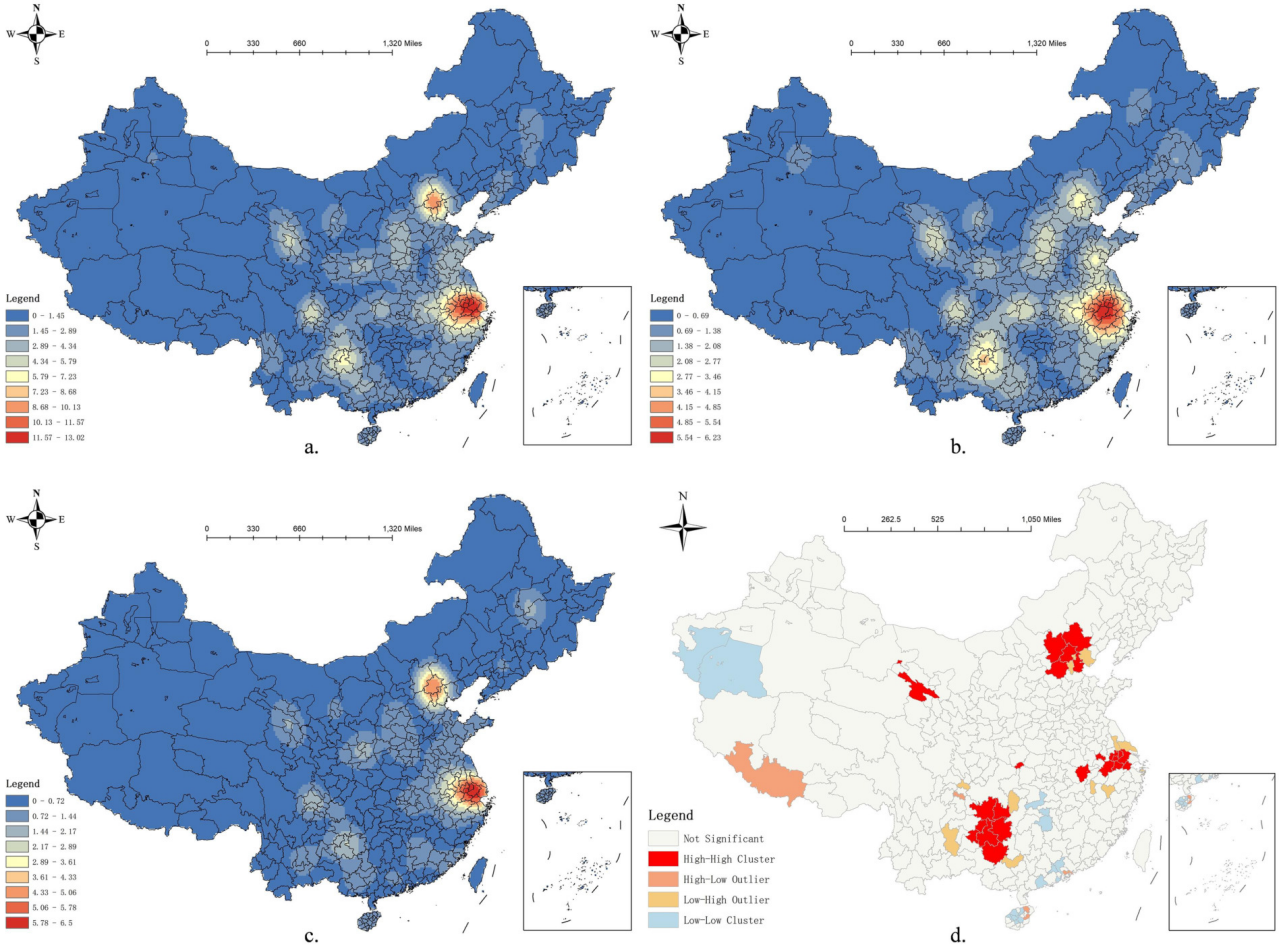
2024 Overall kernel density map of STPs **(a)**; kernel density map of natural STPs **(b)**; kernel density map of cultural STPs **(c)**; local spatial autocorr lation map **(d)**.

Overall, the spatial distribution of STPs in China exhibits the characteristics of “two core regions and multiple distinctive regions” (see Figure 2a). The two core regions are the Yangtze River Delta urban agglomeration centred around Shanghai, Suzhou, Jiaxing, Huzhou, Changzhou, and Wuxi, and Beijing–Tianjin–Hebei urban agglomeration centred around Beijing, Tianjin, and Langfang. The Yangtze River Delta region has a high degree of openness, developed economy, and prosperity, providing a strong economic foundation for the development of sports tourism (12). Additionally, this region has a dense population and high level of urbanisation, forming a large potential consumer base (50). Local governments have strongly supported the development of sports tourism by investing in the construction of sports facilities and hosting sports events. The Beijing–Tianjin–Hebei region has a solid economic foundation, dense population, and developed service industry. Beijing, which is the capital of China, has rich historical and cultural resources that attract global tourists (31). Simultaneously, as the “Dual Olympic City”, Beijing has abundant sports resources, which not only enhance the influence of sports tourism but also strengthen the regional sports culture atmosphere.

By comparing the kernel density of STPs in these two regions, one can see that the Yangtze River Delta region extends westward from the central urban agglomeration to Lu'an and Anqing, and southward to Quzhou, while the Beijing–Tianjin–Hebei region only radiates from Beijing to its surrounding cities. Therefore, Shanghai, as the leading city in the Yangtze River Delta region, has a much stronger influence on the development of the sports tourism industry than Beijing.

The multiple distinctive regions include the Chengdu–Chongqing urban agglomeration centred around Chengdu; Central Guizhou urban agglomeration centred around Guiyang, Anshun, and Qiannan; Buyi People and Miao People Autonomous Prefecture; and Lanzhou–Xining urban agglomeration centred around Xining and Haidong.

The kernel densities of the different types of STPs exhibit little regional variation with no significant overall differences; however, each type has its own density characteristics. From a national perspective, the density of natural STPs (Figure 2b) is generally higher than that of cultural STPs (Figure 2c). Natural STPs develop in multiple places, whereas cultural STPs are relatively more concentrated.

In addition to the urban agglomerations mentioned above, regions with high natural STP density include the southern part of Shandong Province, centred around Linyi, Shennongjia Forestry District in Hubei Province, and most parts of Shanxi Province. These regions have rich and well-known natural landscapes, including Mount Tai and Yimeng Mountain in southern Shandong, the Shennongjia Forestry District in western Hubei, and Lüliang Mountains and Taihang Mountains in Shanxi.

However, as shown in Figure 2c, the density of cultural STPs is notably higher in the Beijing–Tianjin–Hebei and Yangtze River Delta urban agglomerations than in other regions. In this study, cultural STPs are defined as sports events, destinations, and specialised tourism routes that are primarily rooted in historical traditions, cultural practices, and social institutions, rather than being dependent on natural landscapes. This spatial pattern can be interpreted from the perspective of agglomeration economies, where regions with higher levels of economic development tend to attract and concentrate on related industries, talent, infrastructure, and institutional support, creating positive externalities that enhance efficiency and reduce operational costs. Consequently, cultural STPs cluster in these urban agglomerations, benefiting from dense networks of service providers, cultural institutions, and consumer demand, which collectively facilitate project formation, coordination, and sustainable operations.

### 3.3 Spatial autocorrelation analysis of STPs in China

The spatial distribution of STPs in China is highly variable, which Beijing, Shanghai, Suzhou, and Chongqing having 36, 24, 20, and 21 STPs, respectively, ranking as the top four cities. However, 22.31% of the analysed cities have no resources. The spatial distribution of STPs varies significantly among municipalities, making it necessary to conduct a spatial autocorrelation analysis.

Using the ArcGIS 10.8 software, the overall Moran's *I* of the STPs was calculated. The results reveal that the global Moran's *I* is 0.1621, *Z*-score is 4.9251, and *p*-value is 0.00. The *Z*-score is greater than 2.58 and passes the significance test at a 0.01 level, indicating that the distribution of STPs in China in prefecture-level cities exhibits significant clustering characteristics.

To explore whether the clustered regions exhibit high- or low-value clustering characteristics, the Getis–Ord General G index was calculated. The results revealed that the observed value of the General G was 0.0038, expected value was 0.0026, *Z*-score was 6.0066, and *p*-value was 0.00. The observed value of the General G was greater than the expected value, *Z*-score was greater than 2.58, and it passed the significance test at the 0.01 level, indicating that the distribution of STPs in prefecture-level cities in China generally exhibits high-value clustering characteristics.

To observe the clustering traits among municipalities in a more intuitive manner, ArcGIS 10.8 was employed to compute the Anselin Local Moran's *I*.

Four types of spatial associations are typically identified in the local indicators of spatial association results. A “High–High Cluster” indicates areas with high values surrounded by neighbouring units that also exhibit high values, reflecting the strong spatial agglomeration of high-level attributes. Conversely, a “Low–Low Cluster” refers to low-value areas surrounded by other low-value areas, suggesting the spatial clustering of disadvantage or weak development. A “High–Low Outlier” represents a high-value unit surrounded by low-value neighbours, highlighting a spatial anomaly or localised concentration. Similarly, a “Low–High Outlier” corresponds to a low-value unit embedded within high-value surroundings, indicating spatial heterogeneity. These patterns reveal the spatial dependence and heterogeneity of STPs across prefecture-level cities in China.

Figure 2d reveals the presence of both high- and low-value clustering traits among the municipalities. Areas characterised by high-value clustering are predominantly found in the urban agglomerations of Beijing–Tianjin–Hebei, the Yangtze River Delta, Central Guizhou, and Lanzhou–Xining. Low-value clustering areas are mainly concentrated in western Xinjiang, Hunan, Guangdong, and Hainan. Overall, the local clustering features of STPs in China exhibit more high-value clustering and less low-value clustering, and most regions do not exhibit obvious clustering characteristics.

### 3.4 Analysis of the factors influencing the spatial distribution of China's STPs

Based on the geographic interaction theory of human–land relationships, STPs can be considered as cultural products arising from the interplay between natural environments and human activities. Therefore, their spatial formation and distribution are influenced by multiple factors, including ecological endowments, socioeconomic development, industrial structure, transportation accessibility, policy, and institutional support. To capture these dynamics systematically and draw on recent empirical evidence, this study selects indicators across six key dimensions: population, economy, industry, transportation, finance, and environment.

Higher population density fosters economies of scale and market demand, which underpin the clustering of sports tourism resources, as supported by studies demonstrating the significant influence of population and GDP on resource distribution pat-terns (7).

Indicators such as regional GDP and investment capacity reflect the ability to attract and sustain sports tourism, which is consistent with the finding that economic prosperity and financial inputs significantly drive spatial resource patterns (11).

The strength of related tertiary sectors (e.g., culture, leisure, and hospitality) supports STP development, aligning with analyses that classify industrial support and market cultivation among the top influencing factors (32).

Accessibility is a key enabler of tourism and transportation capacity notably influences spatial distribution, as revealed by detector-based analyses (33).

These indicators were analysed using the factor detector, enabling the quantification of each variable's explanatory power and identification of the dominant drivers of spatial patterns across China. Indicator details are listed in Table 1.

**Table 1:** Factors influencing the spatial distribution of STPs in China.

Dimension	Indicator	Unit
Population	Permanent resident population	10,000 persons
	Urbanisation rate	%
	Number of students enrolled in higher-education institutions	Persons
Economy	Per capita GDP	Yuan
	GDP growth rate	%
Industry	Proportion of tertiary industry	%
	Operating income of tertiary industry	100 million Yuan
Transportation	Total road mileage	km
	Road density	km/km²
	Total public transport passenger volume	10,000 persons
Finance	General public budget expenditure	100 million Yuan
	Balance of deposits in financial institutions	100 million Yuan
Environment	Proportion of days with good air quality	%
	Green space area	Hectares

In contrast to other studies at the provincial level, this study analyses prefecture-level cities. The advantages of prefecture-level city analysis are as follows. (1) Prefecture-level cities are more detailed and operational than provincial-level cities. (2) Prefecture-level cities are more closely related to the local socioeconomic environment, allowing for the more precise identification of factors influencing the spatial distribution of STPs. (3) Prefecture-level cities allow for specific policies tailored to the development and protection of local STPs.

The collected data were classified into five categories using the natural breakpoint method in ArcGIS 10.8. Fourteen indicators were selected as independent variables and the number of STPs in each city was used as the dependent variable. A geographic detector program was used for factor detection. The results in Table 2 reveal that the 10 indicators of permanent resident population, urbanisation rate, number of students enrolled in higher education institutions, per capita GDP, proportion of tertiary industry, operating income of the tertiary industry, total public transport passenger volume, general public budget expenditure, balance of deposits in financial institutions, and green space area passed the significance test, indicating that the spatial distribution of STPs in China is mainly influenced by population, economy, industry, transportation, finance, and environment characteristics.

**Table 2:** Factor detector results for the factors influencing the spatial distribution of STPs in China.

Indicator	*q*-value	*p*-value
Permanent resident population	0.3012	0.00
Urbanisation rate	0.1430	0.00
Number of students enrolled in higher education institutions	0.2961	0.00
Per capita GDP	0.1437	0.00
GDP growth rate	0.0151	0.36
Proportion of tertiary industry	0.1625	0.00
Operating income of the tertiary industry	0.4010	0.00
Total road mileage	0.0358	0.47
Road density	0.0239	0.13
Total public transport passenger volume	0.3894	0.00
General public budget expenditure	0.4217	0.00
Balance of deposits in financial institutions	0.4581	0.00
Proportion of days with good air quality	0.0262	0.12
Green space area	0.2689	0.00

**Figure 3:**
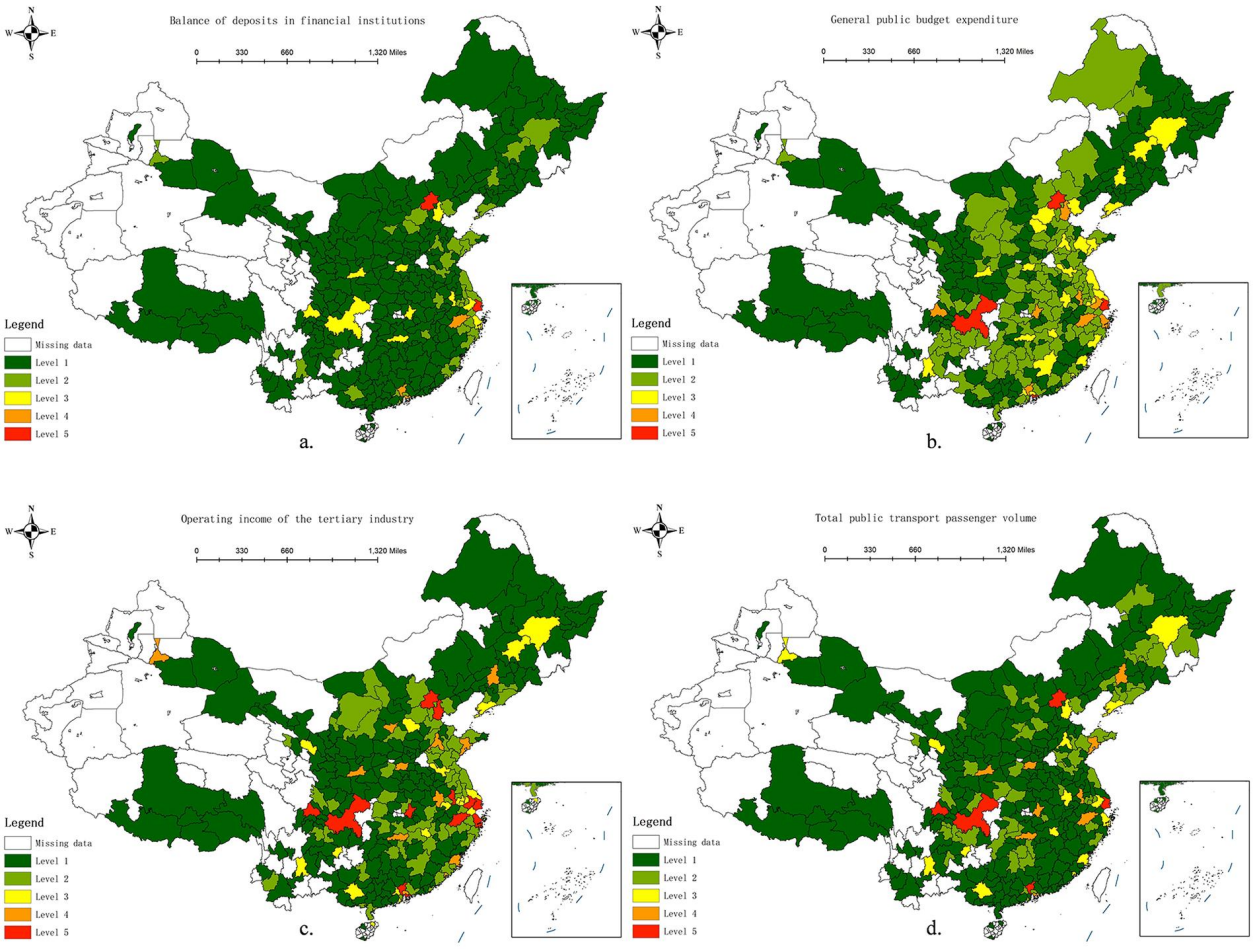
Spatial stratification of dominant factors identified by the Geodetector. Balance of deposits in financial institutions **(a)**. General public budget expenditure **(b)**. Operating income of the tertiary industry **(c)**. Total public transport passenger volume **(d)**.

To enhance the spatial interpretability of the Geodetector results, spatial stratification maps were generated for the four dominant factors with the highest *q*-values (see Figure 3). Each factor was classified into five levels using the natural breaks method, consistent with the stratification applied in the Geodetector analysis. The spatial patterns of these factors show clear correspondence with the clustering of sports tourism projects, providing visual support for their strong explanatory power. These maps do not directly represent *q*-values but illustrate the spatial heterogeneity underlying the detected factor effects.

Regarding the *q*-value, which reflects the degree of influence, the balance of deposits in financial institutions ranks first (0.4581), followed by general public budget expenditure (0.4217) and the operating income of the tertiary industry (0.4010). These results indicate that the spatial distribution of STPs is primarily driven by economic and fiscal conditions, particularly the concentration of financial resources, government fiscal capacity, and tertiary industry development level. This trend suggests that sports tourism is not only dependent on natural endowments or cultural resources but is also closely linked to regional economic strength and industrial structure.

Four indicators, namely GDP growth rate, total road mileage, road density, and proportion of days with good air quality, did not pass the significance test.

## 4 Discussion

### 4.1 Characteristics of sports tourism agglomeration

The spatial distribution of STPs in China exhibits pronounced heterogeneity, shaped by the combined effects of natural resource endowments, economic development, and policy–institutional support (32). Urban agglomerations with favourable physical environments, strong economic foundations, and coordinated governance frameworks tend to form high-density clusters of STPs.

Natural landscapes constitute the fundamental resource base for adventure- and ecology-oriented sports tourism (34). Regions such as the Chengdu–Chongqing, Central Guizhou, and Lanzhou–Xining urban agglomerations benefit from diverse landforms, including mountains, plateaus, forests, rivers, and grasslands, which provide suitable venues for outdoor and experiential sports activities (35). In addition, rich ethnic traditions and historical cultures—such as Bashu culture (36) and minority festival sports—enhance the cultural attractiveness of sports tourism and support the development of region-specific sporting events (37). Improved transportation infrastructure and ecological conservation further strengthen accessibility and environmental sustainability, facilitating the expansion of sports tourism in these regions (38).

In contrast, culture-oriented STPs are more concentrated in economically developed urban agglomerations such as Beijing–Tianjin–Hebei and the Yangtze River Delta. High levels of economic development provide financial resources, advanced infrastructure, and institutional capacity for hosting large-scale sporting events, preserving cultural heritage, and promoting sports tourism brands (33, 39). Economic prosperity thus acts as a primary driver of cultural STP clustering by reinforcing investment, innovation, and market visibility (40).

Policy coordination and institutional integration play a critical role in amplifying these spatial patterns (41). Cross-regional development plans, joint event hosting mechanisms, resource-sharing platforms, and talent exchange systems—implemented across major urban agglomerations—have promoted the integrated development of sports and tourism industries. These policy frameworks enhance regional cooperation, optimise resource allocation, and strengthen the clustering effect of STPs.

Overall, the spatial agglomeration of sports tourism in China results from the interaction between natural suitability, economic capacity, and coordinated policy intervention. Natural endowments determine development orientation, economic strength reinforces clustering intensity, and institutional support provides a stable framework for sustainable and integrated growth.

### 4.2 Discussion of core driving factors and peripheral factors

The balance of deposits in financial institutions serves as a critical proxy for the overall financial capacity and investment potential of a region. From a resource allocation perspective, regions with higher deposit balances generally demonstrate stronger capital accumulation, more diversified financial markets, and higher investment willingness, which provide the necessary financial guarantees for STP development (42). The establishment and operation of STPs, whether based on nature or culture, require substantial capital input for infrastructure construction, facility maintenance, event organisation, and marketing. Without adequate financial resources, regions with rich natural or cultural endowments may face significant constraints when transforming these advantages into competitive tourism products (31).

General public budget expenditure, as a core tool for local government resource distribution (43), directly affects the geographical distribution, type, structure, and service levels of STPs through structural investments, political guidance, and spatial planning.

Local government fiscal budgets activate the “space production” process described by Lefebvre (44) through targeted investment in specific regions. For example, the construction of large sports venues, distinctive sports parks, and waterfront sports belts often relies on fiscal appropriations for spatial restructuring. Such projects form resource aggregation effects through the core–periphery model, shaping the core nodes of regional sports tourism.

Tertiary industry operating income is a critical indicator of regional economic vitality, particularly in sectors directly related to services, culture, and tourism. In the context of sports tourism, higher tertiary industry revenues reflect a robust service economy capable of supporting the development, management, and operation of STPs, including accommodation, catering, entertainment, and event services. Empirical studies indicate that regions with strong tertiary industry performance tend to host a greater number and diversity of tourism projects because service sector capacity facilitates both the supply of tourism experiences and aggregation of demand (45).

Although the GDP growth rate is a widely used indicator of economic performance, it fails to explain the spatial distribution of STPs in China. The GDP growth rate reflects short-term economic fluctuations, whereas sports tourism development, which encompasses planning, infrastructure, and marketing, requires long-term financial accumulation and structural support.

The total road mileage and road density indicators did not pass the significance test, indicating that regions with lower road densities may still have many STPs. It reflects changes in travel patterns and accessibility preferences. This finding is consistent with that of Li and Chen (46), who reported that high-speed rail positively impacts urban tourism.

Regarding the proportion of days with good air quality, Figure 2c, which presents the kernel density of cultural STPs, provides interesting insights. Economically developed regions host a greater number of cultural STPs; however, owing to their higher population density, more vehicles, and more intensive industrial activities, the air quality in these regions is often suboptimal. However, this pattern does not imply that environmental factors are unimportant for sports tourism. For example, indicators such as green spaces still play a significant role in shaping the distribution of tourism projects. Greenspace systems not only improve the urban ecological environment but also provide important spaces for sports tourism (40).

### 4.3 Innovation and limitations

The innovation of this study is reflected in three main aspects.

First, this study constructs a comprehensive and long-term spatial database of STPs across 296 prefecture-level cities in mainland China. By systematically integrating POI data over multiple time periods, the study provides a fine-grained representation of the temporal evolution and spatial expansion of sports tourism at the national scale, which remains insufficiently explored in existing research.

Second, this study introduces an integrated analytical framework that combines GIS-based spatial analysis with the Geodetector model to examine the clustering characteristics and driving mechanisms of sports tourism. Unlike previous studies that mainly focus on descriptive spatial patterns, this approach explicitly reveals spatial heterogeneity and quantitatively identifies the explanatory power of multiple influencing factors, thereby offering a mechanism-oriented interpretation of sports tourism agglomeration.

Third, the study distinguishes the differentiated roles of natural, socioeconomic, and infrastructural factors in shaping sports tourism clustering across urban agglomerations. By visualising the spatial stratification of dominant factors, the research provides intuitive spatial evidence supporting the Geodetector results and generates targeted policy implications for region-specific and sustainable sports tourism development.

Despite the depth of the analysis presented in this paper, some limitations should be acknowledged. First, the data used in this study mainly focused on officially recognised and reported STPs, which may not fully capture all potential resources in China. Future research should consider incorporating more comprehensive and updated data sources to provide a more comprehensive picture of the spatial distribution of STPs. Second, our analysis was conducted primarily at the city level. Although this approach provides a more detailed understanding of local variations, it may not fully capture broader regional and national trends. Future research should further explore grading and evaluation systems to reflect the scale and comparability of different types of STPs. Finally, this study is constrained by data availability. Some potentially relevant variables, such as residents' disposable income, policy support intensity, and sports facility density, could not be included due to the lack of consistent city-level statistics. Future studies may incorporate these indicators when more comprehensive data become available.

### 4.4 Policy recommendations

Based on the findings of this study, we propose several policy measures to promote the more balanced and sustainable development of STPs across China.

Promote balanced regional development. To mitigate structural disparities, national and local governments should strengthen fiscal transfers and target investments in regions with low STP densities such as Tibet, Xinjiang, and Qinghai. Supporting infrastructure development, particularly in transportation and public services, can enhance accessibility and reduce the barriers to project implementation.

Leverage unique regional advantages. Western and ecologically fragile regions should prioritise eco-sports tourism and culturally embedded projects that build upon their distinctive landscapes and ethnic traditions. By fostering specialised tourism niches, these regions can create competitive advantages while preserving their ecological sustainability.

Strengthen financial and institutional support. Given the significant role of financial resources and fiscal expenditure in driving the distribution of STPs, policies should encourage diversified financing models, including public–private partnerships, digital financial inclusion, and dedicated funds for sports tourism innovation.

Enhance the integration of sports and urban development. Urban agglomerations such as the Yangtze River Delta and Beijing–Tianjin–Hebei should continue to optimise the coordination of sports and tourism industries but with greater emphasis on spillover mechanisms that benefit surrounding underdeveloped areas.

Embed sustainability principles. Policymakers should ensure that new STPs incorporate ecological protection, community participation, and long-term operational sustainability. This approach requires planning frameworks that balance economic growth with environmental stewardship and cultural preservation.

## 5 Conclusion

This study comprehensively analysed the spatial distribution characteristics and influencing factors of STPs in China from a geographical perspective using methodologies such as Kernel Density Estimation, spatial autocorrelation analysis, and geographic detection. Our findings provide several key insights that contribute to the existing body of knowledge and have significant implications for practice and policy.

We found that the spatial distribution of STPs in China is heterogeneous, with higher concentrations in the eastern and southern regions, and relative scarcity in the western and northern regions, which is closely related to the “Tengchong–Heihe Line”, highlighting the influence of population distribution, urbanisation, and economic development on the distribution of STPs.

From an academic perspective, this study extends sports tourism research by introducing a mechanism-oriented spatial analytical framework that moves beyond descriptive distribution analysis. The integration of POI data and the Geodetector model offers a replicable approach for examining spatial heterogeneity and driving forces in tourism-related studies.

In practical terms, the findings provide valuable implications for differentiated and sustainable sports tourism development. Policymakers should formulate region-specific strategies that align with local natural resources, economic foundations, and infrastructure conditions, rather than adopting uniform development models. Such targeted approaches can enhance the efficiency of resource allocation and promote the high-quality development of sports tourism across regions.

## Data Availability

The original contributions presented in the study are included in the article/Supplementary Material, further inquiries can be directed to the corresponding author.
